# Renal clearance of heparin-binding protein and elimination during renal replacement therapy: Studies in ICU patients and healthy volunteers

**DOI:** 10.1371/journal.pone.0221813

**Published:** 2019-08-29

**Authors:** Line Samuelsson, Jonas Tydén, Heiko Herwald, Magnus Hultin, Jakob Walldén, Ingrid Steinvall, Folke Sjöberg, Joakim Johansson

**Affiliations:** 1 Department of Surgical and Perioperative Sciences, Anesthesiology and Intensive Care Medicine (Östersund), Umeå University, Umeå, Sweden; 2 Department of Clinical Sciences, Lund University, Lund, Sweden; 3 Department of Surgical and Perioperative Sciences, Anesthesiology and Intensive Care Medicine, Umeå University, Umeå, Sweden; 4 Department of Surgical and Perioperative Sciences, Anesthesiology and Intensive Care Medicine (Sundsvall) Umeå University, Umeå, Sweden; 5 Department of Hand Surgery, Plastic Surgery and Burns and Department of Clinical and Experimental Medicine, Linköping University, Linköping, Sweden; 6 Department of Anesthesiology and Intensive Care, Linköping University, Linköping, Sweden; University of Notre Dame Australia, AUSTRALIA

## Abstract

**Background:**

Heparin-binding protein (HBP) is released by neutrophils upon activation, and elevated plasma levels are seen in inflammatory states like sepsis, shock, cardiac arrest, and burns. However, little is known about the elimination of HBP. We wanted to study renal clearance of HBP in healthy individuals and in burn patients in intensive care units (ICUs). We also wished to examine the levels of HBP in the effluent of renal replacement circuits in ICU patients undergoing continuous renal replacement therapy (CRRT).

**Methods:**

We measured plasma and urine levels of HBP and urine flow rate in 8 healthy individuals and 20 patients in a burn ICU. In 32 patients on CRRT, we measured levels of HBP in plasma and in the effluent of the CRRT circuit.

**Results:**

Renal clearance of HBP (median (IQR) ml/min) was 0.19 (0.08–0.33) in healthy individuals and 0.30 (0.01–1.04) in burn ICU patients. In ICU patients with cystatin C levels exceeding 1.44 mg/l, clearance was 0.45 (0.15–2.81), and in patients with cystatin C below 1.44 mg/l clearance was lower 0.28 (0.14–0.55) (p = 0.04). Starting CRRT did not significantly alter plasma levels of HBP (p = 0.14), and the median HBP level in the effluent on CRRT was 9.1 ng/ml (IQR 7.8–14.4 ng/ml).

**Conclusion:**

In healthy individuals and critically ill burn patients, renal clearance of HBP is low. It is increased when renal function is impaired. Starting CRRT in critically ill patients does not alter plasma levels of HBP significantly, but HBP can be found in the effluent.

It seems unlikely that impaired kidney function needs to be considered when interpreting concentrations of HBP in previous studies. Starting CRRT does not appear to be an effective way of reducing HBP concentrations.

## Background

Heparin-binding protein (HBP), also known as azurocidin or CAP-37, is a 29 kDa protein found in granulocytes. HBP is released together with other vesicle-stored proteins when neutrophils are activated[1–3]. HBP has several properties. It is antimicrobial, chemoattractant and has the ability to increase vascular permeability facilitating extravasation of leukocytes[4].

A large number of clinical studies have been done in critically ill patients where increased concentrations of HBP have been demonstrated in patients with respiratory failure[5–7], sepsis[8], shock[9], and burns[10]. HBP has also been shown to be associated with the presence and development of acute kidney injury in sepsis[11–13] and mortality[7]. Several studies of patients in the emergency department have evaluated HBP as a biomarker for development of organ failure associated with sepsis[14–16]. While results are promising, the use of HBP as biomarker has not yet been incorporated in routine clinical practice.

Despite an increasing number of studies on HBP, there are no published data on renal elimination of HBP.

It has previously been shown that hemofiltration can influence levels of inflammatory mediators in plasma[17], and we also know that inflammatory responses are induced by the introduction of artificial membranes in extracorporeal blood circuits[18]. There are however, no published data on whether HBP levels in plasma are affected by the hemofiltration or if HBP can be found in the effluent from hemofiltration.

Our aim was to study levels of HBP in plasma and urine/effluent flow in three different contexts–healthy individuals as reference and two groups of critically ill patients previously shown to have increased plasma concentrations of HBP, burn intensive care unit (ICU) patients with different degrees of kidney dysfunction, and critically ill patients during the initiation of continuous renal replacement therapy (CRRT).

## Materials and methods

### Ethical approval

The Regional Ethics Review Board in Linköping approved the burn ICU study (dnr M210-08, head of committee Swan) and the CRRT study (dnr 2010/427-31, head of committee Dahlstedt). The Regional Ethics Review Board in Umeå approved the study in healthy volunteers (dnr 2015/474-31, head of committee Jacobaeus). Healthy volunteers gave written consent. For ICU patients, oral consent was given by the patient or next of kin if the patient was not able due to disease severity. This was approved by the ethics review board. Consent was documented in each patient’s study protocol by the including physician.

### Healthy volunteers

The study was prospective and consisted of eight healthy volunteers. Inclusion criteria were self-reported good health and a negative urinary dipstick. Urine was collected during 8 hours. Urine volume was measured, and a sample was drawn from the collected volume. To verify that plasma levels of HBP were stable, eight blood samples were drawn hourly from one volunteer, four samples were drawn from two volunteers, and two samples were drawn from the other five volunteers, one in the morning and one in the afternoon. For each participant, the mean plasma level of HBP was used for calculating clearance.

Renal clearance was calculated as $Cl=\frac{C_{u}\;x\;V_{u}}{C_{p}}$, where C_u_ is the concentration of HBP in the urine (ng/ml), V_u_ is the urinary volume flow (ml/min) and C_p_ is the plasma concentration of HBP (ng/ml). This equation reflects the elimination of HBP in urine and does not consider reabsorption or secretion in the tubule.

### Burn ICU patients

The study was conducted at a national burn center (Linköping University Hospital Burn Centre) in a cohort of patients that has previously been reported[19]. Twenty patients with burns involving 20% or more of the total body surface area were included. Patients were treated in accordance with a standardized protocol [20], including the Parkland formula for early resuscitation, ventilator treatment when needed, early enteral nutrition, and early excision and grafting of the burn wound, starting within 1–2 days.

Plasma and urine samples were collected, and urine output was recorded twice the first day and then once on the third, sixth, and eighth day after arrival at the hospital. The corresponding times for urine volume collection were approximately 3 and 8 hours the first day and 24 hours in the following days. Both HBP and cystatin C levels were analyzed in the plasma samples, while only HBP levels were analyzed in the urine samples. Cystatin C is emerging as a potential marker of impaired renal function in burn patients[21, 22], and was measured using immune-turbidimetric assay at Linköping University Hospitals laboratory. The level of the upper reference interval at the university hospital laboratory (1.44 mg/l) was used as the cut-off for impaired renal function.

### CRRT patients

This was a prospective, observational study in the 8-bed mixed ICU of Östersund Hospital, a 300-bed hospital in Sweden. During the period 2012–2016, we included 32 adult patients (18 years and over) who started CRRT in the ICU. Patients transferred from another ICU were excluded. Written and oral information was given to the patient or close relative before obtaining consent. Simplified acute physiology scores (SAPS) 3 were recorded on admission. Vascular access with a double lumen catheter using the Seldinger technique was established in all 32 patients. The CRRT equipment used was the Baxter/Gambro Prisma. Continuous veno-venous hemodiafiltration was performed with either an ST150 or Oxiris AN69 hollow-fiber hemofilter using citrate as the anticoagulant. The AN69 membrane used in both filters has an in vivo cut-off value of 35 to 49 kDa. Five ST150 filters and six Oxiris filters were pretreated with heparin when priming the circuit. All of the patients underwent continuous veno-venous hemodiafiltration with an effluent flow of 30–35 ml/kg/h and a blood flow of 160–200 ml/min.

Plasma samples were drawn when CRRT was started and at the time of changing the first and second drainage bag. Samples from the second drainage bag were collected, and the total volume in the drainage bags was measured.

### Handling of samples for the HBP analysis

Blood samples were collected in ethylenediamine-tetra-acetic acid (EDTA) tubes, brought directly to the laboratory, centrifuged, and stored at −80°C until analysis.

Urine samples were collected and immediately brought to the laboratory. In the study with healthy volunteers, the urine was analyzed with a urinary dipstick and under a microscope to rule out the presence of white blood cells. All samples intended for HBP analysis were stored at −80°C.

The concentration of HBP in the samples was measured as described previously[1]. In short, this involved a sandwich ELISA using a monoclonal and polyclonal antibody against human HBP. The technique has intra- and inter assay variabilities of less than 10%.

### Statistics

Descriptive data are presented as medians with interquartile ranges. Because the data were not normally distributed, the Mann–Whitney U-test or the Wilcoxon signed rank test was used for comparison between groups as appropriate. Spearman’s non-parametric correlation coefficient was used to assess the correlation between HBP levels in plasma and in the CRRT effluent. All data were analyzed using SPSS (IBM SPSS Statistics for Macintosh, Version 23.0, Armonk, NY: IBM Corp), and p-values less than 0.05 were considered significant.

## Results

### Healthy volunteers (n = 8)

Eight healthy males aged 38 to 46 years were included in this study. The median plasma concentration of HBP was 7.28 ng/ml (IQR 6.65–8.35 ng/ml), the median urine concentration was 0.87 ng/ml (IQR 0.68–0.94 ng/ml), and the median urine flow was 1.64 ml/min (IQR 0.87–2.73 ml/min). The calculated median HBP clearance was 0.19 ml/min (IQR 0.08–0.33 ml/min).

### Burn ICU patients (n = 20)

Details of the patients are presented in Table 1. The median plasma level of HBP was 14.7 ng/ml (IQR 12.2–21.4 ng/ml), the median urine concentration of HBP was 2.44 ng/ml (IQR 1.59–7.86 ng/ml), the median urine flow was 1.38 ml/min (IQR 0.96–2.43) and the median HBP clearance was 0.30 ml/min (IQR 0.01–1.04 ml/min).

**Table 1:** Characteristics of patients.

	Healthy volunteers	Burn ICU patients		ICU patients on CRRT	
n	8	20		31	
Female n (%)	0 (0)	2 (10)		12 (39)	
Age	43 (39–46)	43 (27–63)		65 (37–83)	
		Type of injury		Main diagnosis:	
		Flame, n (%)	17 (85)	Sepsis, n (%)	17 (53)
		Electrical, n (%)	2 (10)	Intoxication, n (%)	1 (6)
		Scald, n (%)	1 (5)	Lactic acidosis, n (%)	2 (12)
		TBSA %	40 (25–52)	Rhabdomyolysis, n (%)	3 (18)
		ICU LOS, days	64 (23–94)	Liver failure, n (%)	2 (12)
		ICU mortality, n (%)	4 (20)	Chronic kidney failure, n (%)	1 (6)
		30-day mortality,n (%)	4 (20)	Cardiac arrest/heart failure, n (%)	4 (24)
				Ruptured aorta, n (%)	1 (6)
				SAPS III-score	66 (41–85)

Data are presented as numbers and % or as medians and interquartile ranges as appropriate. ICU (intensive care unit); TBSA % (percentage total body surface area); LOS (length of stay). CRRT (continuous renal replacement therapy); SAPS (sequential organ failure assessment).

In patients with plasma cystatin C > 1.44 mg/l, the median plasma level of HBP was 14.5 ng/ml (IQR 11.6–21.6 ng/ml), the median urine concentration of HBP was 3.16 ng/ml (IQR 1.70–46.9 ng/ml), the median urine flow was 1.51 ml/min (IQR 0.99–2.56) and the median HBP clearance was 0.45 ml/min (IQR 0.15–2.81 ml/min).

In patients with plasma cystatin C ≤ 1.44 mg/l, the median plasma level of HBP was 14.8 ng/ml (IQR 12.4–21.9 ng/ml), the median urine concentration of HBP was 2.37 ng/ml (IQR 1.44–4.60 ng/ml), the median urine flow was 1.31 ml/min (IQR 0.94–2.20) and the median HBP clearance was 0.28 ml/min (0.14–0.55 ml/min).

**Fig 1:**
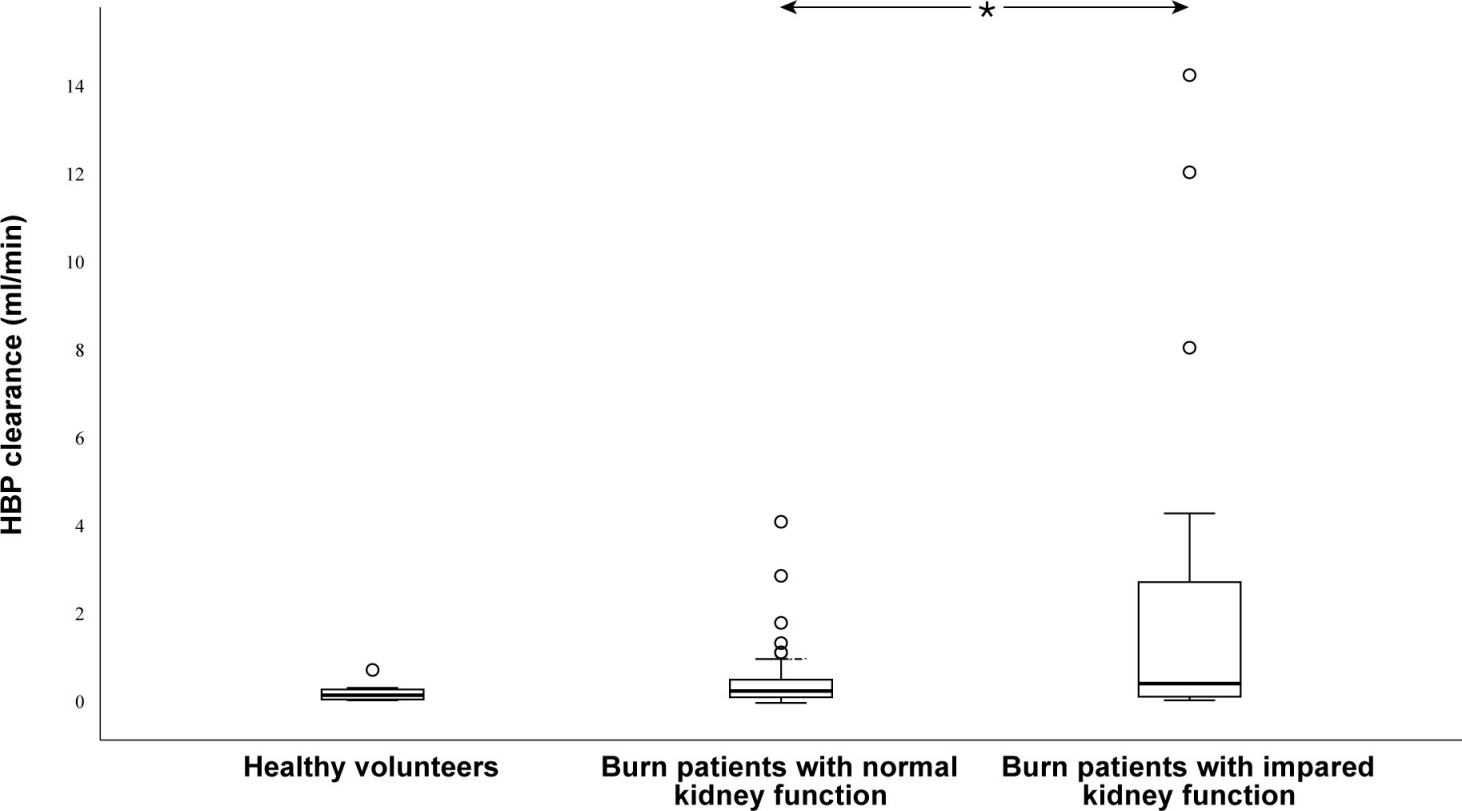
Clearance of heparin binding protein (HBP) in healthy volunteers and burn ICU patients with cystatin C ≤ 1.44 or > 1.44. Boxes indicate the second to third quartile with the median. Brackets indicate min-max values. Circles indicate outliers. * indicates p < 0.05. Clearance was significantly higher in burn ICU patients with cystatin C > 1.44.

HBP clearance was significantly higher in patients with cystatin C > 1.44 mg/l (p = 0.04). There were no significant differences in clearance of HBP between healthy volunteers and burn patients with cystatin C ≤ 1.44 mg/l (p = 0.35) (Fig 1).

### CRRT patients (n = 31)

**Fig 2:**
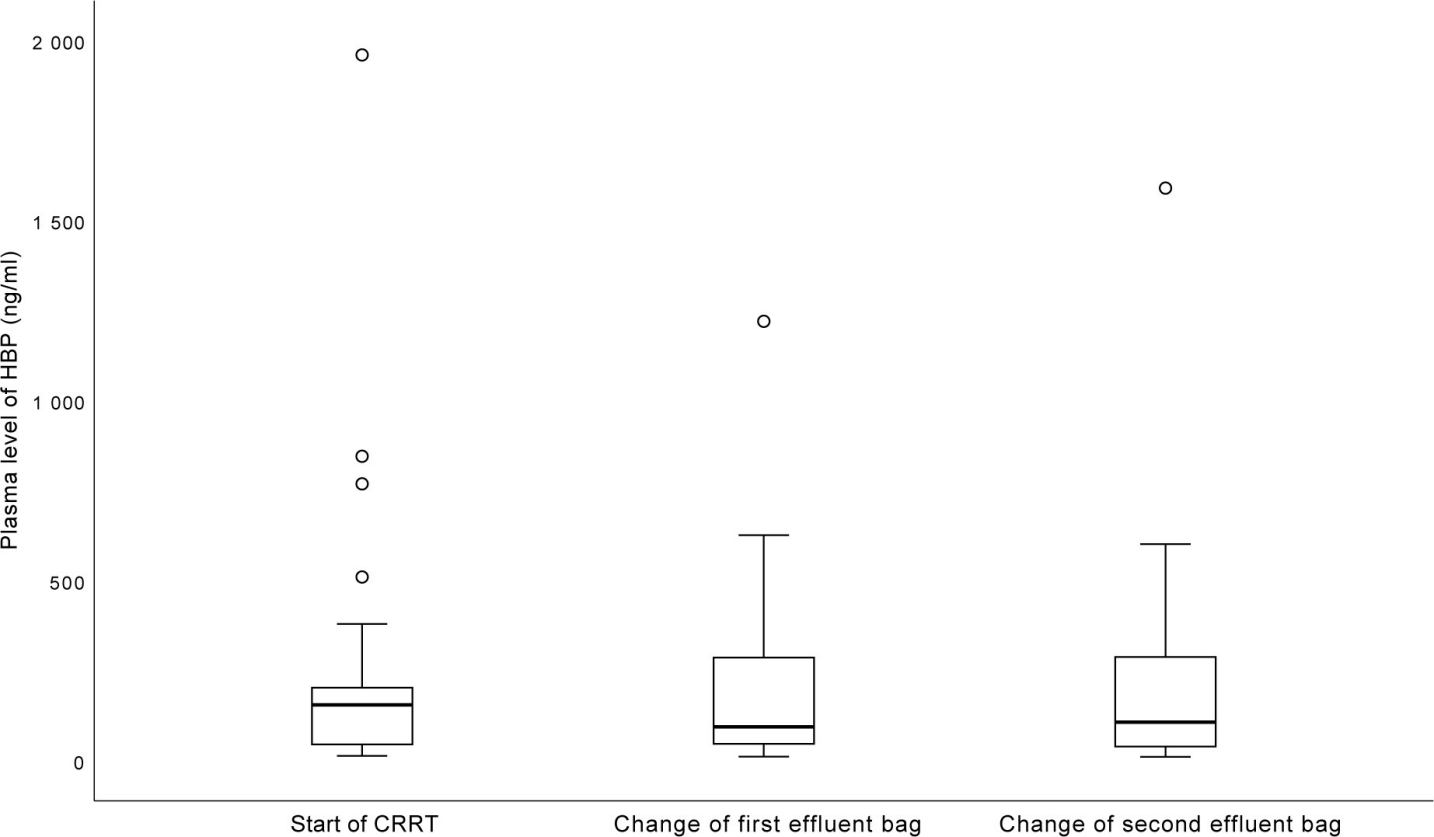
Plasma levels of heparin binding protein (HBP) after the start of CRRT. Boxes indicate the second to third quartile with the median. Brackets indicate min-max values. Circles indicate outliers. There was no significant difference between first and last registration (p = 0.14).

Details of the patients are presented in Table 1. The median plasma level of HBP at the start of CRRT was 155.4 ng/ml (IQR 45.3–220.6 ng/ml). At the time of changing the first drainage bag, it was 91.3 ng/ml (IQR 47.3–287.0 ng/ml), and at the time of changing the second drainage bag it was 98.3 ng/ml (IQR 39.8–288.5 ng/ml) (Fig 2). The time from start of CRRT to changing the second drainage bag ranged from three to six hours. There was no statistically significant increase or decrease in plasma levels of HBP between the first and last sampling time (p = 0.14). The time between changing drainage bags ranged between 60 and 170 minutes. Subdividing the groups according to type of filter or pre-treatment with heparin did not change this result (data shown in S1 Datasets).

**Fig 3:**
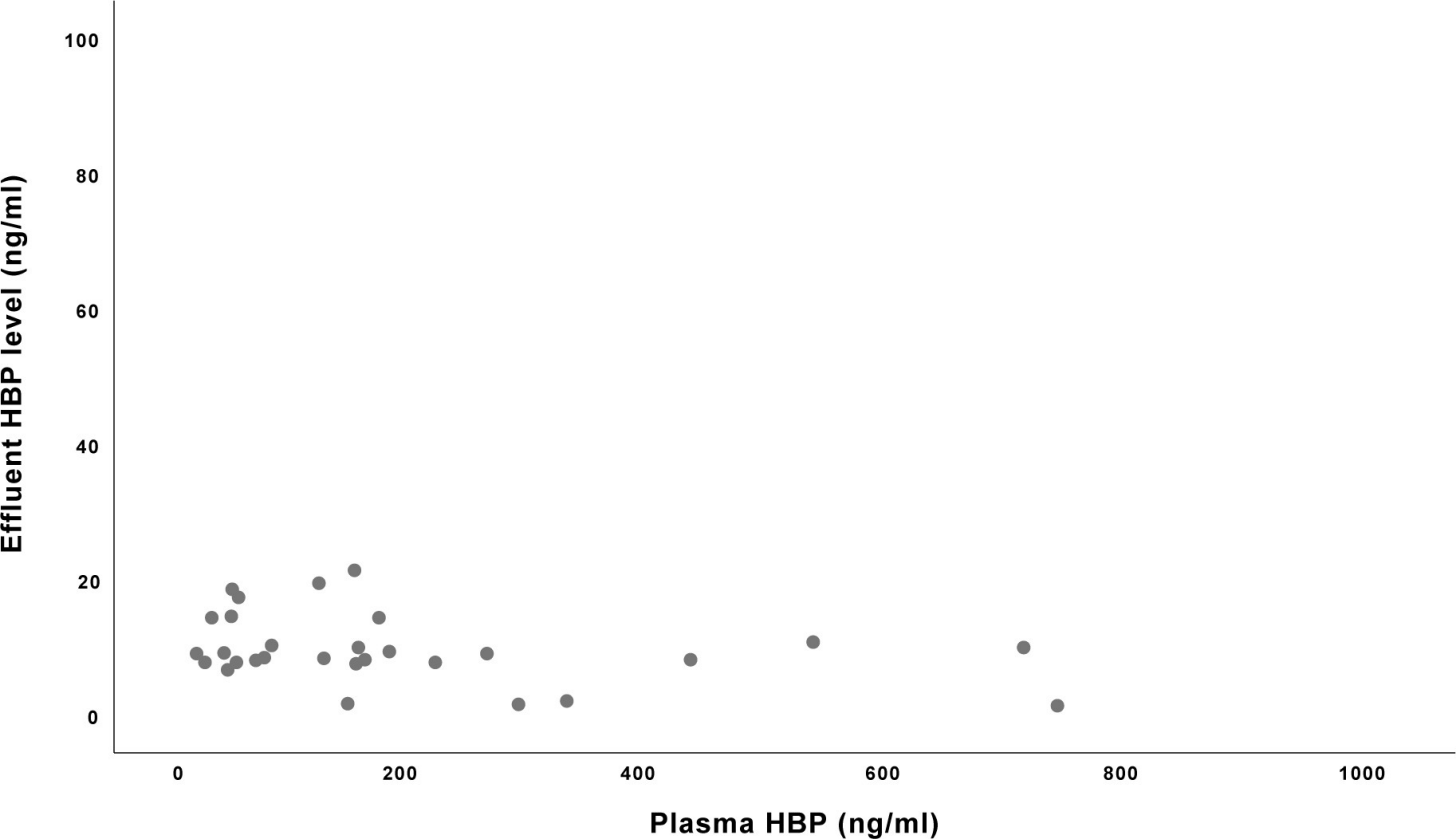
Concentration of heparin binding protein (HBP) in the effluent of CRRT in relation to plasma.

The median concentration of HBP in the effluent was 9.1 ng/ml (IQR 7.8–14.4), and the concentration of HBP in the effluent relative to plasma concentrations is shown in Fig 3.

There was no correlation between the concentration of HBP in the effluent and the concentration of HBP in plasma (rho = −0.14, p = 0.45).

## Discussion

HBP is emerging as a possible biomarker of organ failure associated with sepsis and in critically ill patients. In the evaluation of potential biomarkers, it is important to understand the kinetic properties of the biomarker in the context where it is intended to be used.

We wanted to investigate renal clearance of HBP in patients cared for in a burn ICU and elimination in the effluent from critically ill patients when starting CRRT, both groups who have previously been shown to have increased concentrations of HBP. As a reference we also wanted to investigate renal clearance of HBP in healthy volunteers.

### Healthy volunteers

In this study we showed low levels of HBP in the urine of healthy volunteers, and the levels were lower than what was measured in the control group in a previous study on urinary tract infections[23]. Considering that HBP is a low-molecular weight protein of 29 kDa, the results seem reasonable. In general, low molecular weight proteins are freely filtered in the glomeruli and almost completely reabsorbed by proximal tubular cells[24]. With intact tubule cells, the reabsorption will maintain a low concentration of HBP in the urine. The results of this study imply that there are either other ways of eliminating HBP or that the metabolism of the free pool of HBP is low, and this will be an interesting topic for future studies.

### Burn ICU patients

We showed that the rates of calculated HBP clearance in burn patients with normal levels of cystatin C were not significantly different to those of healthy volunteers. In patients with increased cystatin C, we found significantly higher renal clearance of HBP. Urine concentrations of low molecular weight proteins depend on glomerular filtration and tubular reabsorption, and dysfunction in either or both entities of the kidney will alter the concentration, which might be an explanation for the results presented here.

Activated leukocytes in the urinary tract can release HBP and thereby increase the urinary concentration of HBP, as has been described in patients with cystitis and pyelonephritis[23, 25]. In our study of healthy volunteers, we analyzed the urine with urine dipsticks and microscopy to rule out these sources of HBP. However, in the burn ICU patients, the presence of leukocytes in the urine was not analyzed and might be a possible source of error in our clearance calculations.

While the HBP clearance was significantly higher when renal function was impaired, the clearance was still low. Thus, it seems unlikely that renal function needs to be considered (within reasonable limits) when evaluating HBP levels in plasma from previous or future studies.

### CRRT patients

In ICU patients on CRRT, we demonstrated the presence of HBP in the effluent, and the concentrations of HBP appeared relatively constant despite large variations in plasma concentrations of HBP. Convective removal of a solute depends on transmembrane pressure, on the molecular weight and structure of the solute, and the cut-off point of the membrane. For the AN69 membrane, the average cut-off is 35–40 kDa, which would permit passage of solutes like HBP (29 kDa) and cytokines. De Vries et al. [11] investigated the relative contribution of membrane adsorption and convection on cytokine removal and found that membrane adsorption represented the main clearance mechanism for cytokines. Other studies on inflammatory cytokines have shown elimination by convection as well as by adsorption to the filter [20, 21].

Our findings of relatively constant HBP concentrations in the effluent despite variations in plasma concentrations would suggest convection rather than diffusion. However, we did not look at HBP adsorption to filters, which is a limitation to our study. We did not see any statistically significant consistent increase or decrease in plasma levels of HBP when starting CRRT. An increase in plasma levels due to an inflammatory response to blood being exposed to the CRRT circuits would have seemed just as reasonable as a decrease due to removal by adsorption and convection.

Given the association and possible causal relationship between high levels of HBP in plasma and organ failure and mortality[4, 6–9, 11, 12, 26], the possibility to reduce HBP levels might seem appealing. However, our results suggest that starting CRRT with standard settings and standard filters is not an effective way of reducing these levels.

## Conclusions

In healthy individuals, the calculated renal clearance of HBP is low and is at the same level as in critically ill burn patients with normal kidney function. In critically ill burn patients with impaired kidney function, calculated renal clearance of HBP is increased.

Starting CRRT in critically ill patients does not consistently increase or decrease plasma levels of HBP, but HBP is found in the effluent while on CRRT.

## Supporting information

S1 Datasets Datasets on healthy volunteers, burn ICU patients and patients on continuous dialysis. (XLSX)
